# Role of folate receptor α in the partial rejuvenation of dentate gyrus cells: Improvement of cognitive function in 21-month-old aged mice

**DOI:** 10.1038/s41598-024-57095-x

**Published:** 2024-03-22

**Authors:** A. Antón-Fernández, R. Cuadros, R. Peinado-Cahuchola, F. Hernández, Jesús Avila

**Affiliations:** 1 Centro de Biología Molecular Severo Ochoa, CSIC/UAM, Universidad Autónoma de Madrid, Cantoblanco, 28049 Madrid, Spain; 2 Center for Networked Biomedical Research on Neurodegenerative Diseases (CIBERNED), Madrid, Spain

**Keywords:** Cellular neuroscience, Neural ageing, Transporters in the nervous system

## Abstract

Neuronal aging may be, in part, related to a change in DNA methylation. Thus, methyl donors, like folate and methionine, may play a role in cognitive changes associated to neuronal aging. To test the role of these metabolites, we performed stereotaxic microinjection of these molecules into the dentate gyrus (DG) of aged mice (an average age of 21 month). Folate, but not S-Adenosyl-Methionine (SAM), enhances cognition in aged mice. In the presence of folate, we observed partial rejuvenation of DG cells, characterized by the expression of juvenile genes or reorganization of extracellular matrix. Here, we have also tried to identify the mechanism independent of DNA methylation, that involve folate effects on cognition. Our analyses indicated that folate binds to folate receptor α (FRα) and, upon folate binding, FRα is transported to cell nucleus, where it is acting as transcription factor for expressing genes like SOX2 or GluN2B. In this work, we report that a FRα binding peptide also replicates the folate effect on cognition, in aged mice. Our data suggest that such effect is not sex-dependent. Thus, we propose the use of this peptide to improve cognition since it lacks of folate-mediated side effects. The use of synthetic FRα binding peptides emerge as a future strategy for the study of brain rejuvenation.

## Introduction

Aged cells are rejuvenated through partial reprogramming without the loss of somatic identity^1^. Such reprogramming can be achieved by the cycle expression of the so-called Yamanaka Factors (YF)^2,3^. The expression of these transcription factors brings about an amelioration of some hallmarks of aging in peripheral tissues^4–6^. Despite of some differences in the effect of YF in cells from peripheral tissues and neurons^7^, also, YF promote partial reprogramming (rejuvenation) in neuronal cells in the hippocampal region of aged mice, thereby facilitating cognitive improvement^8^.

Here we tested a strategy to rejuvenate aged brain cells through specific partial reprogramming^9^ using small simple compounds^10^ instead of YF. In this regard, the compounds may reprogram neurons in the brains of aged mice by: (a) increasing adult hippocampal neurogenesis (AHN); (b) inducing epigenetic changes (methylation) in histones or DNA^8,11^; or (c) acting as transcription factors^8,12^. Thus, here we tested whether small compounds replicate the effects of YF on aged neurons. Our findings show that the compounds had no significant impact on AHN. Regarding the second point, YF control epigenetic changes like those linked to the regulation of histone and DNA methylation^5,8^. Changes in the latter sustain the development of the epigenetic clock, which may predict biological (chronological) age^13^. Histone and DNA methylation involve methyl donors and methyltransferases^14^. The universal methyl donor is methionine but other factors like folate could be involved indirectly^14^. YF and their related Thompson factors^11^ increase the activity of DNA methyltransferases like the DNA methyltransferase (DNMT) family^15–17^. Given that DNA methylation can increase in the presence of methyltransferases, through the presence of methyl-donors like the universal methyl-donor (methionine) or folate, here we studied whether the presence of any of these small compounds could replicate the effects of YF in the amelioration of aging features in the dentate gyrus (DG)^8^. Our results suggest that the presence of folate is enough to increase cognition in aged mice. Furthermore, folate can act on cells by binding to folate receptors, folate receptor α (FRα) being the most studied receptor, and which, in addition, serves as a transcriptional factor^18^. Thus, our results indicate that folate induces the transcriptional activity of FRα. Also, we tested the capacity of a synthetic FRα-binding peptide^19^ to mimic the effect of folate on this factor. Indeed, the injection of this peptide into the hippocampus of aged mice resulted in cognitive improvement similar to that achieved with folate injections. Given these observations, this FRα-binding peptide emerges as a potential alternative treatment to folate to improve cognition.

## Results

### Partial improvement in the object recognition and Y maze tests of mice treated with folate but not with *S*-adenosyl-methionine (SAM)

**Figure 1:**
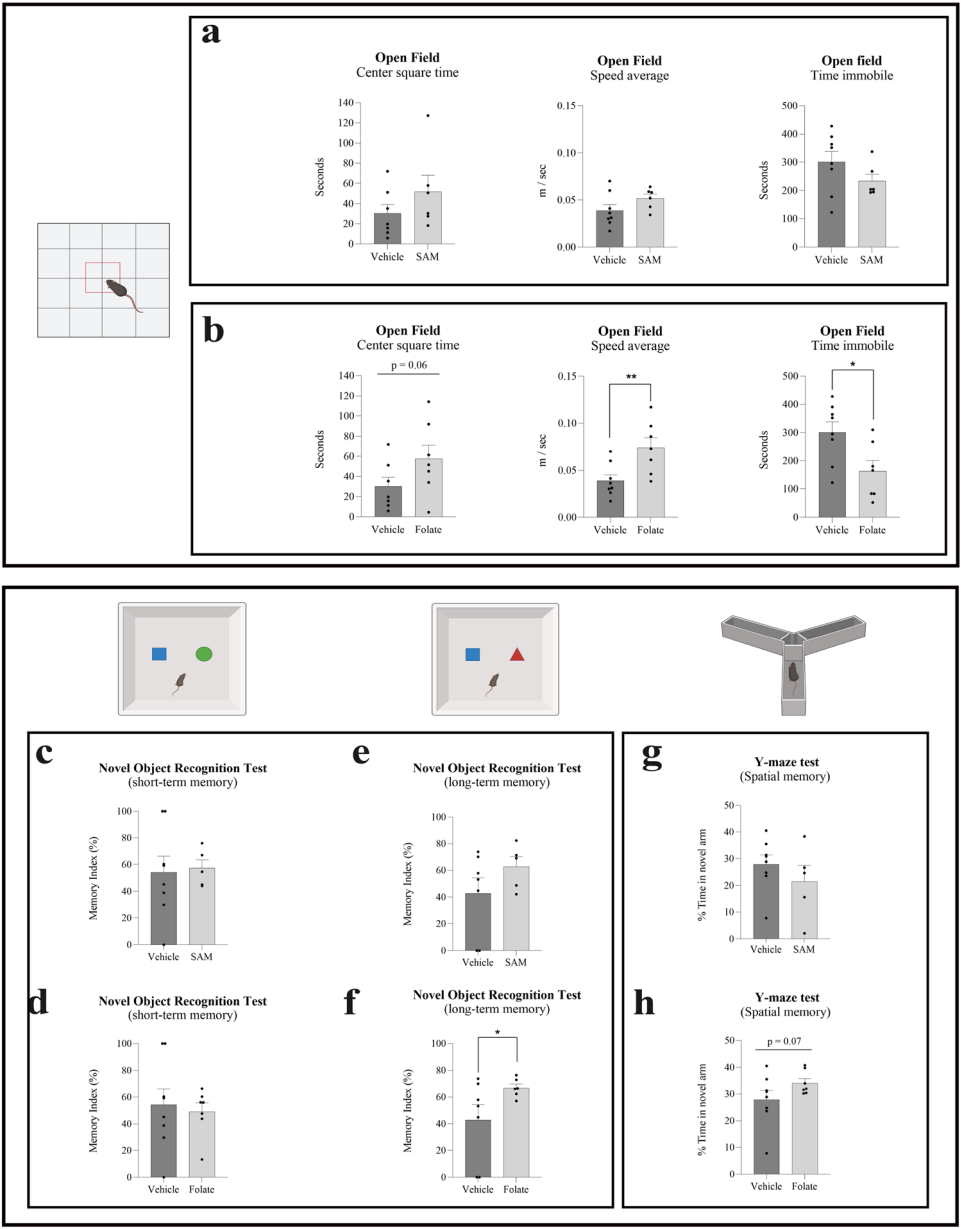
Effect of methyl donors, SAM and folate, on behavior. Aged wild-type mice receiving a single dose of folate via hippocampal infusion showed behavioral changes when compared with mice treated with vehicle solution. Such changes were not observed in animals receiving *S*-adenosylmethionine (SAM). (**a,b**) Histograms show some of the main data from the open field test: time spent by mice in the center square of the box, average speed of movement, and time spent immobile. Folate-treated mice showed a tendency for improvement (t = 1.661; df = 12) in anxiety-depression-like behavior (indicated by the time spent in the center square), as well as in general motor activity (illustrated by the rest of the histograms; t = 2.928, df = 13 and t = 2.590 df = 13). (**c–h**) Histograms show results from two memory tests, namely the novel object recognition test (NOR) for short- [(**c,d**); t = 0.1925, df = 11 and t = 0.3578, df = 13, respectively] and long-term recognition memory [(**e,f**) t = 1.302, df = 10 and t = 1.863, df = 13, respectively], and the Y maze test (t = 0.9982, df = 11 and t = 1.540, df = 11, respectively) for spatial memory (**g,h**). Significant differences were observed between folate-treated mice and the vehicle-treated group (**f,h**), but none were found between SAM-treated and vehicle-treated mice in any memory test (**c,e,g**). Black bars represent the mean ± SEM of vehicle-treated groups and gray bars the mean ± SEM of SAM/Folate-treated groups. All data were analyzed by Student's t-test. *p < 0.05, **p < 0.01.

SAM, folate, or vehicle was intracranially injected (single dose) into the dorsal hippocampal hilar region (SI 1) of the mice. The effects of these injections on performance in the behavioral tests were analyzed one week later (see “Methods”). In the first round of surgeries, we screened SAM treatment in a group of 14 mice: n = 6 mice treated with SAM at an average age of 20.4 months (5 male and 1 female) and n = 8 mice treated with vehicle at an average age of 19.7 months (4 male and 4 female). SAM injection had no effect on behavioral performance in the Open Field (OF) (Fig. 1a) or memory (Fig. 1c, e, g) tests. However, mice treated with folate (n = 7, 1 male and 6 female, at an average age of 19.6 months; Fig. 1b, d, f, h) showed an improvement in locomotor activity, distance traveled, average speed, time spent immobile, and anxiety-depression-like (time spent in the central square) behavior compared to vehicle-treated mice (n = 8, 4 male and 4 female, at an average age of 19.7 months). Figure 1f, h show the differences in long-term memory (5 days) in the novel object recognition and spatial short-term memory.

The result of a single injection of folate was similar to that reported for aged mice expressing YF in a cyclical manner^8^. Folate improved the long-term memory (5 days) post-familiarization with the novel object (Fig. 1f), but no significant differences were found for short-term (2 h) memory (Fig. 1d). In addition, folate-treated mice showed a strong tendency to have a better spatial memory compare to the aged controls, as reflected by their performance in the Y maze test (Fig. 1h). Our observations indicate that folate injection in dentate gyrus affected mouse behavior.

### Possible cellular effects of folate on hippocampal region

The effects of folate behind cognitive improvement could be related to the following: (a) an increase in adult hippocampal neurogenesis (AHN); (b) epigenetic changes in histones or DNA; or (c) the induction of transcription factors. In addition, several biochemical outputs of folate metabolism (SI 2) that are not covered in this work could produce some side effects on cells.

First, we tested the effect of folate on AHN (SI a–d), studying changes in brain lipid-binding protein (blbp) (SI 3a) and doublecortin-immunoreactive (SI 3d) cell densities. Also, to label dividing cells, mice were injected intraperitoneally with CldU a week before perfusion, thus allowing the study of newborn 1-week-old cells in the SGZ. A single injection of folate into the hippocampus led to only a subtle increase in newborn CldU-ir cells (SI 3b) and in 1-week-old neurons (Dcx and CldU double-positive cells) (SI 3c) in the SGZ. We found no differences in Blbp-ir (SI 3a) or doublecortin-ir cell densities (SI 3d) upon folate injection. Therefore, folate had no significant effect on adult hippocampal neurogenesis (AHN).

The expression of aging-related genes correlates, as suggested by the heterochromatin loss aging model^20^, with increasing alterations of chromatin located in heterochromatin regions. In this regard, we examined whether folate injection modifies the degree of histone methylation, namely at positions H3K9me3 and H4K20me3, which are present mainly in heterochromatin regions. The presence of folate 14 days post-injection did not modify the level of methylation at H3K9me3 (SI 4a, b) or H4K20me3 (SI 4c, d).

Our findings suggest that a single injection of folate might not be enough to promote reliable changes in histone methylation levels. Therefore, we tested whether folate affects DNA methylation (SI5). Thus, significant increases in 5mC were observed in the granular neuronal layer (P = 0.0132) of the DG where the injection was performed (SI 5c) and it also found a lower increase at CA3 (P-value = 0.05) and CA1 (P-value = 0.06) (SI 5d, e). Regarding 5hmC, we also tested its levels, since the accumulation of 5hmC in postmitotic neurons is related to “demethylation” (5mC → 5hmC), thus facilitating gene expression. No significant changes were found in hippocampal neuronal layers (SI 6).

These data provide results on the effect of folate on adult neurogenesis at the dentate gyrus and the impact of folate on the methylation of histone and DNA in hippocampal neurons. Next, we focused our study on folate as an inducer of transcription factors.

### The potential of folate as a transcription factor inducer in neuronal human cells through FRα

In addition to acting as a methyl donor, folate can exert additional actions on DNA. Upon binding to folate, FRα^21^ is internalized into the cell nucleus, where it can act as a transcription factor^18^ to facilitate the expression of specific genes (see also SI 2).

To directly test the action of folate in the nuclear internalization of FRα in neuronal cells, we used a neuronal human cell line (SK-N-SH) as a model to confirm the results reported by Boshnjaku et al.^18^. In this regard, we have showed that the presence of folate results in the internalization of FRα into the nucleus (SI 7 a, b) of these neuronal cells in a similar fashion to what occurs in other cell types. In addition, upon neuronal localization of FRα, we found an increase in the expression of the protein Sox2 (SI 7 c, d), one of the YF. This observation supports the findings of a previous report^22^ and could explain the capacity of folate to replace YF and facilitate cell rejuvenation.

Also, the expression of YF like Sox2 have been found to increase the expression of GluN2B in the hippocampus^8^. This expression is compatible with a possible effect of folate in the cognitive improvement found in aged mice, reported herein.

### Effect of hippocampal folate administration on the expression of protein related with juvenile stages

Furthermore, changes in gene expression occur during aging. Some genes expressed in young cells are not expressed in old cells and vice versa. For example, the gene codifying for GluN2B protein is expressed mainly in young neurons^23^, whereas genes involved in the formation of PNN are found mostly in aged tissue^24^. Both neurons and the PNN are involved in hippocampal neuroplasticity mechanisms.

### Changes in GluN2B expression

**Figure 2:**
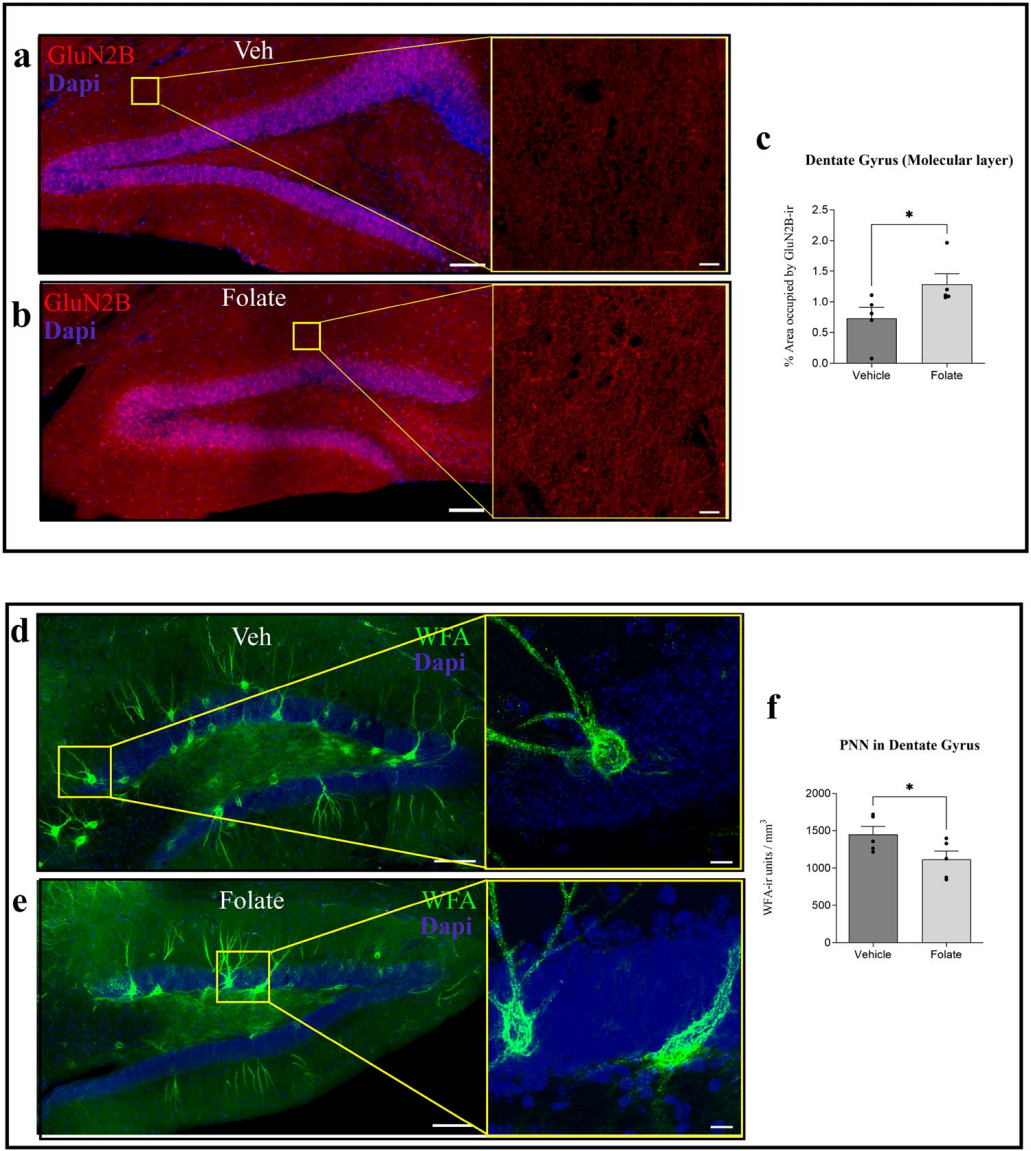
Effect of folate on the expression of NMDA receptor subunit GluN2B and on extracellular matrix organization. Effects of folate on the presence of the NMDA receptor subunit GluN2B in mature hippocampal neurons. (**a,b**) Representative stitched confocal images of GluN2B distribution in the dentate gyrus (DG) of aged wild-type mice treated with vehicle (**a**) or folate (**b**). Higher magnifications (×40  oil immersion objective was used with additional zoom applied) of the molecular cell layer of (**a**,**b**) (yellow squares) are shown. (**c**) Histogram showing the percentage of area occupied by the GluN2B signal in the molecular layer of the DG. The effect of the absence (dark grey) or presence (light gray) of folate is shown. Treatment with folate induced a significant increase in GluN2B expression in the DG (t = 2.269, df = 8). **(d,e)** Representative stitched confocal images of *Wisteria floribunda* agglutinin (WFA, in green), as a marker of perineuronal nets (PNN) in the DG of vehicle-treated and folate-treated mice. The nuclear marker DAPI is shown in blue. Higher magnifications of the molecular cell layer of (**d**,**e**) (yellow squares) are shown. (**f**) Single-dose treatment with folate resulted in statistically significant changes in PNN density in the DG (t = 2.166, df = 8) (mean ± SEM; *p < 0.05, Student’s t-test). Scale bars, 100 µm (**a,b,d,e**) and 10 µm (higher magnifications).

We previously reported^8^ that the presence of YF induces an increase in GluN2B expression in the molecular layer of the DG. GluN2B is one of the subunits of NMDA receptor, which is present mainly in young animals and may be related to greater cognitive capacity^23^. Figure 2 shows the immunoreactivity of GluN2B in the DG (Fig. 2a, b) of mice treated or not with folate. GluN2B immunoreactivity in the molecular layer increased in the former (Fig. 2c).

#### Changes in extracellular matrix organization

The ECM is organized into PNN. These structures are involved in synaptic stabilization in the mature brain^25^. They comprise proteoglycans like neurocan, brevican, aggrecan, phosphacan, and versican, which can be linked to PNN through their binding to hyaluronan^26,27^. PNN can ensheath interneurons, thereby affecting their function^28^. Aging is associated with an increase in PNN in some regions of the brain, including the hippocampus^29,30^. Thus, we checked whether these structures are reorganized in response to folate injection in the hippocampal region (Fig. 2d, e). Folate treatment induced a significant reduction in PNN unit density labeled with WFA lectin in comparison with vehicle-injected counterparts (Fig. 2f).

### What is the main effect of folate on cognitive improvement?

To test whether the effect of folate on cognition is due mainly to changes in DNA methylation or to alterations of transcription modulated by factors like nuclear FRα, we tested the effect of a FRα-binding peptide^19^ to determine whether it can mimic the effect of folate (n = 7, 2 male and 5 female, with average age of 22.6 months for FRα-binding peptide-injected mice and n = 11, 4 male and 7 female, with average age of 21.9 months for vehicle-injected mice). To this end, we synthesized a peptide (CTVRTSAEC) able to bind FRα^19^. Although we took into account that the FRα-binding peptide is not a methyl donor and should facilitate only the transcriptional activity of FRα-binding peptide, we first tested whether the peptide contributes to DNA methylation. No significant changes in 5mC or 5hmC were detected (SI 8).

To test whether the peptide induces changes at the transcriptional level, we included mice in vitro studies using a neuronal human cell culture model, SK-N-SH cells. These cells were used to test the effect of CTVRTSAEC peptide on the expression of specific genes. Binding the peptide to FRα facilitates the internalization of FRα into the cell nucleus, where it may act as a transcription factor (SI 7 a, b), increasing the expression of Sox2 in that cell line (SI 7 c, d).

These results agree well with those previous data^22^ indicated that folate, through FRα, upregulates YF expression like Sox2, but also indicated that the folate effect could be replaced by CTVRTSAEC peptide.

#### Effect of the FRα-binding peptide on Sox2 expression

**Figure 3:**
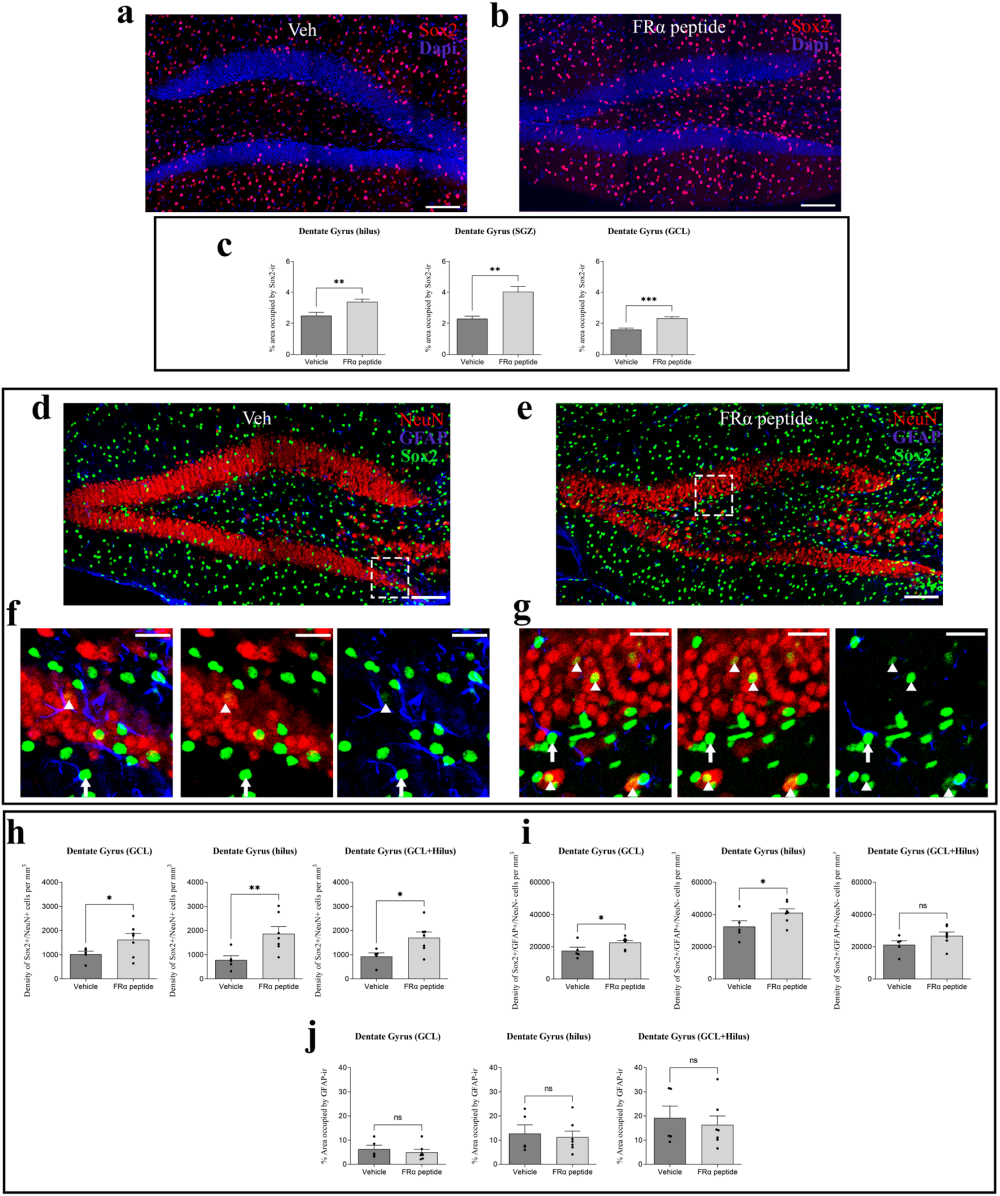
Effect of FRα-binding peptide on the expression of Yamanaka factor Sox2 in dentate gyrus and specifically in neurons. (**a,b**) Representative stitched confocal images of Sox2 expression (in red) in the dentate gyrus (DG) of mice treated with vehicle or FRα-binding peptide. The nuclear marker DAPI is shown in blue. (**c**) Single-dose treatment with FRα-binding peptide resulted in statistically significant changes in Sox2 immunoreactivity expression in the hilus region, subgranular zone (SGZ) and granular cell layer (GCL), of the DG (t = 3.142, df = 9; t = 3.715, df = 9 and t = 4.673, df = 9, respectively in each layer). (**d,e**) Representative stitched confocal images of Sox2 (in green), NeuN (in red) and GFAP (in blue) co-expression in the dentate gyrus (DG). Higher magnifications of the dentate gyrus of (**d,e**) (segmented squares) are shown in f and g respectively. Immunoreactive cells for Sox2 and NeuN (neurons expressing Yamanaka factor Sox2) are marked by a white arrowhead. (**h**) Single-dose treatment with FRα-binding peptide resulted in statistically significant changes in Sox2 immunoreactivity expression in neurons from the hilus region, granular cell layer (GCL) and in the whole DG (t = 2.497, df = 10; t = 2.645, df = 10 and t = 4.220, df = 10, respectively in each layer). (**i**) Single-dose treatment with FRα-binding peptide resulted in statistically significant changes in Sox2 immunoreactivity expression in astrocytes from the hilus region, the granular cell layer and in the whole DG (t = 2.855, df = 10; t = 1.857, df = 10; t = 2.513, df = 10, respectively in each layer). (**j**) Single-dose treatment with FRα-binding peptide did not lead to statistically significant changes in the area occupied by astrocytes (GFAP immunoreactivity) in the hilus region, granular cell layer, and the entire DG (t = 0.3513, df = 10; t = 0.6755, df = 10; t = 0.4631, df = 10, respectively in each layer) (mean ± SEM; *p < 0.05, **p < 0.01, ***p < 0.001; Student’s t-test). Scale bar, 100 µm (**a,b,d,e**) and 25 µm [higher magnifications in (**f,g**)].

As previously indicated, folate facilitates the expression of YF, including Sox2^22^. Thus, we studied whether the FRα-binding peptide also affects the expression of this transcription factor in a mouse model in vivo (Fig. 3a, b). Treatment of aged mice with this peptide resulted in a significant general increase in Sox2 expression in various regions of the DG (Fig. 3c). Considering that Sox2 is not only expressed in neurons but it can be found also in astrocytes, we have tested if neurons, specifically, increased the expression of sox2 after FRα-binding peptide treatment. A triple-immunofluorescence study using astrocyte markers (GFAP) and neuronal markers (NeuN) allowed us to investigate the exclusive colocalization of each Sox2 + cell specifically with the neuronal marker (Fig. 3d-g). The analysis revealed that treatment with the FRα-binding peptide at the DG specifically increased the expression of the Yamanaka factor Sox2 in neurons from all different layers of DG (Fig. 3h). This increase was found also in GFAP-immunoreactive cells, but it was less significant (Fig. 3i). In addition, it is important to note that the total number of astrocytes did not change after peptide treatment (Fig. 3j).

#### Effect of the FRα-binding peptide on GLUN2B expression

**Figure 4:**
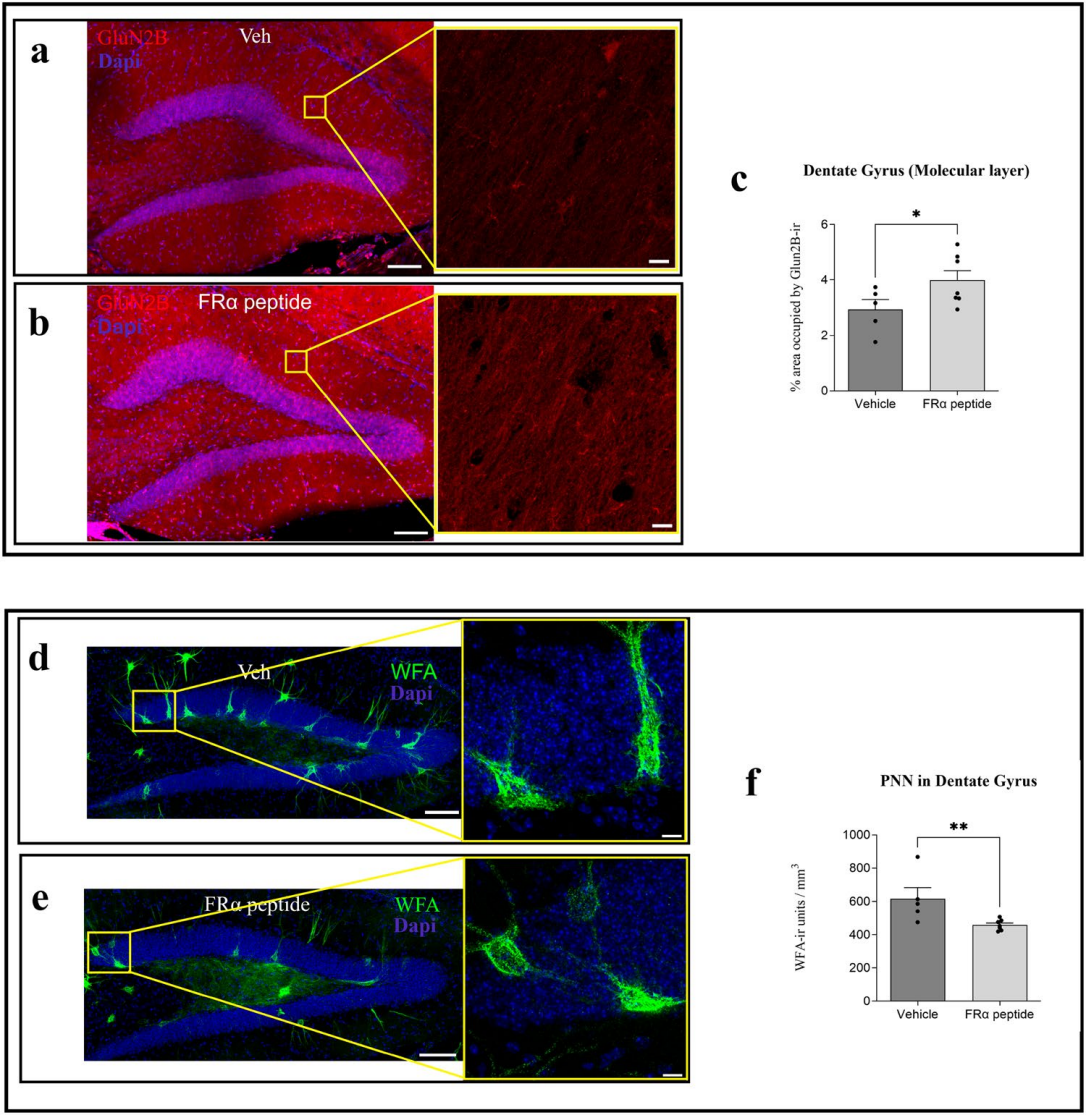
Effect of FRα-binding peptide on the expression of GluN2B expression, and extracellular matrix organization (**a,b**). Representative stitched confocal images of GluN2B distribution in the DG of aged wild-type mice treated with vehicle (**a**) or FRα-binding peptide (**b**). Higher magnifications (×40  oil immersion objective was used with additional zoom applied) of the molecular cell layer of (**a,b**) (yellow squares) are shown. The nuclear marker DAPI is shown in blue. (**c**) Histogram showing the percentage of area occupied by the GluN2B signal in the molecular layer of the DG. The effect of the absence (dark grey) or presence (light gray) of FRα is shown. Treatment with FRα-binding peptide induced a significant increase in GluN2B expression in the DG (t = 2.069, df = 10).** (d,e)** Representative stitched confocal images of Wisteria floribunda agglutinin (WFA, in green), as a marker of perineuronal nets (PNN) in the DG of mice injected with vehicle or FRα-binding peptide. The nuclear marker DAPI is shown in blue. Higher magnifications of the granular cell layer of (**d,e**) (yellow squares) are shown. (**f**) Single-dose treatment with FRα-binding peptide resulted in statistically significant changes in PNN density in the DG (t = 2.771, df = 10) (mean ± SEM; *p < 0.05, **p < 0.01; Student’s t-test). Scale bar, 100 µm (**a,b,d,e**) and 10 µm (higher magnifications).

Given that the presence of YF facilitates the expression of GluN2B^8^, we addressed whether the presence of the FRα-binding peptide, which upregulates the expression of Sox2, also increases the expression of GluN2B in aged mice (Fig. 4a, b). Treatment with this peptide led to an increase in GluN2B expression in the molecular layer of the DG (Fig. 4c), as occurred with folate treatment.

#### Effect of the FRα-binding peptide on extracellular matrix organization

The FRα-binding peptide affected ECM organization in a similar fashion to folate (Fig. 4d, e), thereby reducing the density of PNN in the DG (Fig. 4f).

Given these findings (“Effect of the FRα-binding peptide on GLUN2B expression” and “Effect of the FRα-binding peptide on extracellular matrix organization”), we suggest that neuronal signaling is enhanced through changes in excitatory (glutamatergic)^8,23^ but also in inhibitory neurons^28^. In addition, both results could be related to each other, since alterations in the expression of PNN components during aging could be modulated by alterations of NMDA function^31^.

### Cognitive improvement in the presence of FRα-binding peptide

**Figure 5:**
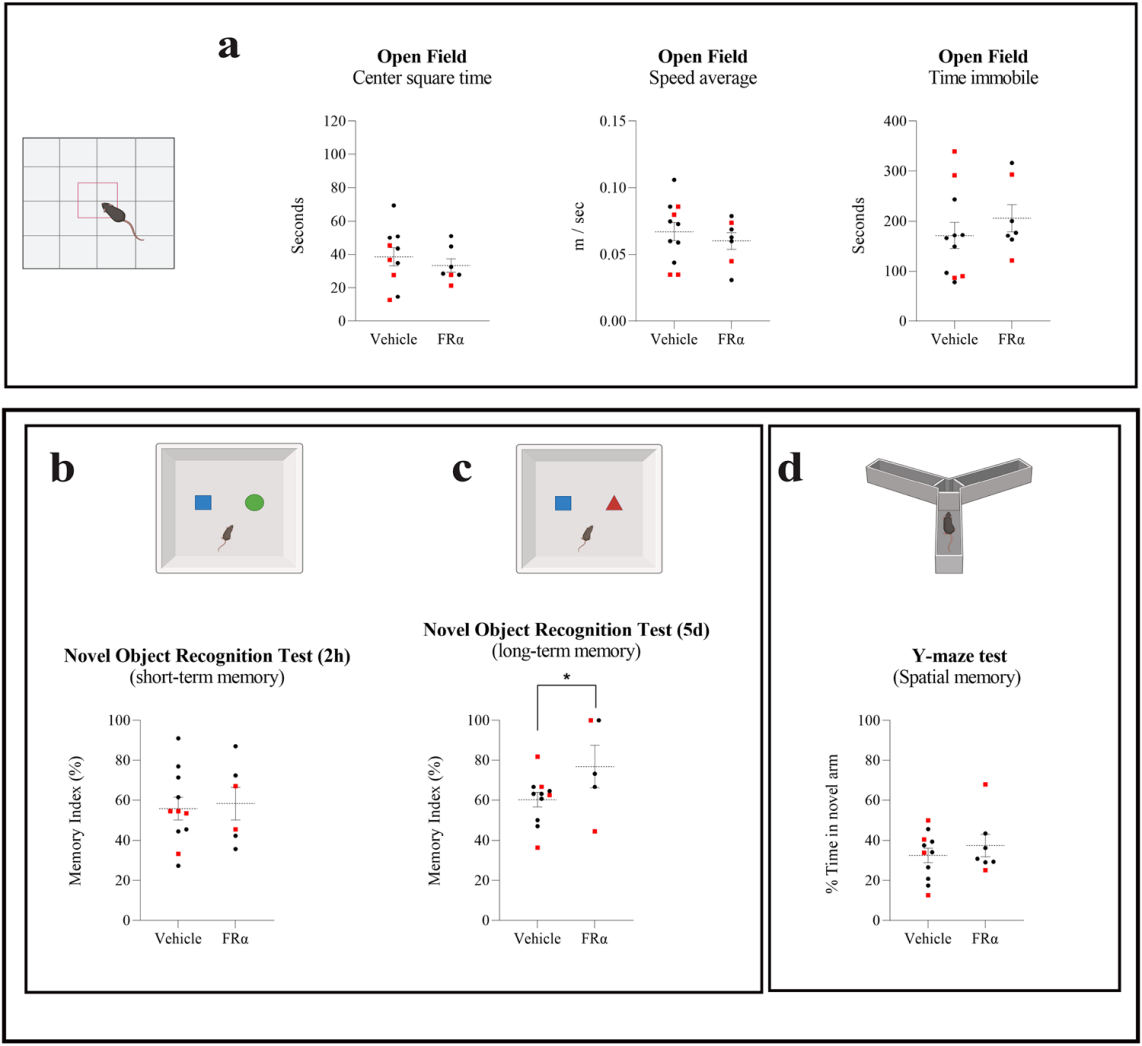
Effect of the FRα-binding peptide on cognition. (**a**) Scatter plots show some of the main data from the open field test in mice treated with FRα-binding peptide: time spent by mice in the center square of the box, average speed of movement, and time spent immobile. No changes were found after FRα-binding peptide treatment in anxiety-depression-like behavior (t = 0.7068, df = 15 for center square time), nor in general motor activity (t = 0.7003, df = 16 for speed average and 0.8766, df = 16 for time immobile). The exclusive analysis of female mice has yielded the same results as the data from mixed-sex groups, with no changes observed in any of the OF test parameters (t = 0.7545, df = 9 for center square time; t = 1.512, df = 10 for speed average and t = 1.010, df = 10 for time immobile). (**b,c,d**) Scatter plots show the results from two memory tests: the novel object recognition (NOR) for short-(2 h) (**b**) and long-term recognition memory (5 days) (**c**) and the Y-maze test for spatial memory (**d**). Mice treated with the peptide showed a significant improvement in memory retention 5 days after the first training session, as shown by memory index of novel object recognition test (t = 1.905, df = 14). Treatment with FRα-binding peptide did not have a significant effect on short-term recognition memory (t = 0.7752, df = 16 in spatial memory test and t = 0.2599, df = 15 in NOR test) but a significant difference was observed in the long-term tests. The exclusive analysis of female mice has yielded the same results as the data from mixed-sex groups, with no changes observed in Y-maze test (t = 0.4131, df = 10) or NOR for short-term recognition memory (t = 0.02451, df = 9), and significant improvement in NOR for long-term recognition memory (P = 0.0132, t = 2.718 and df = 8). Grouped column scatter plot displaying means ± SEM of both the vehicle-treated group and the peptide-treated group are shown. Black plots represent female mice and red square plots represent male mice. All data were analyzed by Student`s t-test. *p < 0.05.

We tested whether treatment with the FRα-binding peptide has a similar effect to that of folate on cognition. Intrahippocampal injection of this peptide under the same conditions as previous folate experiments did not bring about significant changes on behavioral performance in the Open Field (Fig. 5a), short-term memory performance, either in spatial memory or recognition memory, relative to vehicle-treated mice (Fig. 5b–d). However, treatment with this peptide led to a significant long-term improvement of recognition memory, thereby reproducing the same results as those achieved with folate injections (Fig. 5c). To investigate potential sex-related effects, we reanalyzed behavioral and cognitive data considering only the data from females (to have a N > 5). The results revealed no significant changes compared to our previous findings with mixed-sex data, indicating that the effects of the FRα-binding peptide are not dependent on sex (Fig. 5a–d).

## Discussion

Here we devised and tested a novel strategy to rejuvenate the neuronal cells of aged mice. The approach involved a single injection of folate into the hippocampus to reproduced the reported effects of YF on these cells^8^. Folate treatment resulted in the partial rejuvenation of dentate gyrus cells by partial reprogramming. Given our results, this treatment emerges as an alternative to treatment with YF, to be translated to a possible human therapy.

The effect of folate on cognition in aged mice could be attributable to the following: (a) increased adult neurogenesis; (b) epigenetic alterations, like changes in DNA methylation; or (c) changes in transcription that may result in the reorganization of structures of the ECM or in the expression of juvenile genes.

Our results revealed that folate treatment had no effect on AHN. Regarding epigenetic changes related to methylation, they may require methyl donors like folate or methionine (SAM). Although methionine is the universal methyl donor, folate may also act as a donor through folate-methionine metabolism^32^. However, some differences between the presence of methionine and folate can be found. High levels of methionine are toxic for humans^33^. However, this effect has not been reported for folate. Methionine toxicity could be due to the fact that this compound is a precursor of the toxic homocysteine^34^. Also, it should be noted that a high-methionine diet induces Alzheimer disease-like symptoms^35^. Furthermore, methionine can have other side effects, for example, if methionine is converted to SAM, the latter can also re-methylate methionine through homocysteine^32,36^. On the contrary, there is a decrease in the levels of folate and an increase in homocysteine with age^32^. In addition, low levels of folate may correlate with the cognitive decline of elderly patients^37^. In fact, folate supplement has been proposed as a potential treatment for reducing memory deficits^38,39^. Here we tested the effect of folate and SAM on cognitive improvement. Despite the involvement of both compounds as methyl donors, only folate was found to have an impact on cognition, similar to that described for YF^8^.

As indicated, the effect of folate on cognition is not explained by changes in AHN or epigenetic modifications. In this regard, we found that the main action of folate could be through transcription, by regulating the expression of specific genes.

In this context, folate interacts with its cell receptor, FRα. Upon binding to folate, this receptor is transported to the cell nucleus, where it serves as a transcriptional factor^18^. FRα is involved in the expression of the YF Sox2^22^. Also, FRα may induce the expression of genes such as GluN2B (subunit of NMDA receptor, which is considered a juvenile gene^8,40,41^), or may be involved in the regulation of PNN, which is affected by aging^24,30,42^ at regions like the hippocampus^31^.

PNN are a specific type of ECM wrapped around some interneurons^28^ that restrict the plasticity of these cells. PNN are dynamic structures that undergo changes throughout postnatal development, and their reduction has been associated with the facilitation of neuronal plasticity and memory enhancement^27,29,43,44^. In this regard, our results in aged mice suggest that a reduced density of PNN causes the reorganization of the ECM, inducing a more plastic and juvenile-like organization.

Given that folate may have negative side effects and that its effects on cognition are exerted through the transcriptional activity of FRα, we tested the capacity of a synthetic FRα-binding peptide to mimic folate function. The treatment of aged mice with this peptide also improved cognition and thus emerges as a suitable (and better) alternative to folate.

Although folate and FRα-binding peptide share an effect on gene expression, folate, together with its nine derivatives^45^, but not the peptide, is involved in a variety of other biological actions (SI 2). Folate can interact with different receptors and transporters while FRα binding peptide only interacts with FRα, a protein that is abundant in brain, compared with other tissues (Human Protein Atlas. FOLR1: https://www.proteinatlas.org/ENSG00000110195-FOLR1). Examples of folate action include the biosynthesis of methionine by supplying one carbon unit (methyl groups), the biosynthesis of purines and thymidine (related to DNA synthesis), amino acid homeostasis^46,47^, epigenetic maintenance (as methyl donor), and redox defense^48^. Another difference found in this work at behavioral level, was a reduced anxiety-depression-like behavior found in the presence of folate but not in the presence of FRα binding peptide. Our results indicate that the main effect of folate on cognition is not through any of the previous effects as a methyl donor but rather through its action on FRα, which is shared with FRα-binding peptides. In this regard, folate increases the expression of factors like Sox2 and GluN2B or alters the organization of the ECM, specifically PNN, which, in addition, will improve cognitive functions such as long-term memory.

Thus, our current working hypothesis explains the role of folate or the FRα-binding peptide in the cognitive improvement of aged mice, as described in the following pathway: folate or peptide, nuclear FRα, Sox2↑, GluN2B ↑, ECM organization ↓, improved cognition.

In this context, we propose that folate and/or the indicated FRα-binding peptide promote cognition, mainly through the action of nuclear FRα.

In summary, of the three possible effects of folate tested on cognitive improvement, namely increases in (a) adult neurogenesis; (b) DNA methylation, or (c) neuroplasticity by reorganizing ECM structures or enhancing the expression of juvenile genes like GluN2B, we propose that (c) is the main effect exerted by this metabolite on cognitive improvement.

## Conclusions

In conclusion, our results indicate that treatment with folate or FRα-binding peptide induces the rejuvenation of aged DG cells, thereby enhancing cognitive improvement (without an apparent sex-related effect). Mainly, by activating the FRα pathway, these two compounds emerge as a future peptide strategy to treat cerebral aging and age-associated neurodegenerative diseases.

## Methods

### Ethics statements

Animals were housed in accordance with European Community Guidelines (directive 86/609/EEC) and handled following European and local animal care protocols. The animal experiments were approved by the CBMSO Ethics Committee (AECC-CBMSO-13/172) and the National Ethics Committee (PROEX 102.0/21). All methods were performed in accordance with the relevant guidelines and regulations. The study is reported in accordance with ARRIVE guidelines (https://arriveguidelines.org).

### Animals

Animals were housed in a specific pathogen-free facility under standard laboratory conditions. They were housed 4–5 per cage with food and water available ad libitum and maintained in a temperature-controlled environment on a 12/12-h light/dark cycle with light onset at 8 a.m. A total of 39 C57BL/6 wild-type male (16) and female (23) mice were bred in the animal facility at the CBMSO.

### Experimental design

Mice aged 21 months were used for the *S*-(5′-adenosyl)-l-methionine chloride (SAM), folate, and synthetic peptide experiments. The animals were randomly injected in the hilus region of both brain hemispheres (2 µl in each one) with vehicle (phosphate-buffered saline (PBS) or distilled water), or one of three molecules of interest, namely SAM (0.25 mg/ml diluted in water; Sigma, ref: A7007; m.w. 398.5 g/mol), folic acid (0.25 mg/ml diluted in PBS; Sigma, ref: F7876-10G; m.w., 441 g/mol), or a synthetic FRα-binding peptide (5 mg/ml, diluted in PBS; Abyntec; m.w., 1121 g/mol). The pH of SAM and the folic acid solution was adjusted with NaOH to ~ 7 to avoid negative effects caused by an overly acidic medium. One week after the intracerebral injections, the mice were assessed using behavioral (open field trial) and memory tests (novel object recognition and Y maze test) to determine the potential functional effects of the metabolites on the central nervous system (SI 1a). Two weeks after surgery, the mice were perfused immediately after completing the Y maze test.

### Stereotaxic surgery and intrahippocampal microinjection

After anesthesia with isoflurane, the mice were secured in a stereotaxic frame (Kopf). Holes were drilled bilaterally in the skull at the injection sites (one per hemisphere) using a microdrill with a 0.5 mm bit. The following stereotaxic coordinates were used for intrahippocampal injections (from bregma): anterior–posterior −2.0; lateral 1.4; and dorsoventral −2.2 (SI 1b). A 33-gauge needle Hamilton syringe coupled to a syringe pump controller mounted on the stereotaxic frame was used to inject 2 µl of vehicle or metabolites at each site. Injections were given at 0.25 µl/min and after completing the inoculation of the entire solution volume, a 3-min wait was observed before removing the needle from the injection site. Prior to completely removing the needle from the brain tissue, another 3-min pause was implemented halfway through in order to minimize potential damage. After the injections, the skin was sutured and buprenorphine (Sigma, USA) was administered subcutaneously. The animals were allowed to recover for 1 h on a heating pad before being returned to the cage. The animals were treated with ibuprofen (Dalsy in drinking water) for the following 5 days. They remained in the cage for an additional week before the start of behavior tests and were sacrificed 2 weeks after surgery (SI 1a).

### CldU injection protocol

The mice received a total of three injections, spaced every 2 h (200 ul/injection, intraperitoneally in alternative sites of the body) of 5-Chloro-2’-deoxy-Uridine (CldU, Sigma reference C6891; i.p. 42.75 mg/Kg body weight) one week after cranial surgery and microinjections of the solutions, and just 1 week before sacrifice (SI 1a). This approach allowed us to monitor the effects of the treatments on the proliferation of new cells in the subgranular zone (SGZ), a key region of the brain for neurogenesis, labeling one-week-old newly generated cells.

### Animal sacrifice and tissue processing

The mice were anesthetized with an intraperitoneal pentobarbital injection (Dolethal, 60 mg/kg body weight) and transcardially perfused with saline. Brains were separated into two hemispheres. One was removed and fixed in 4% paraformaldehyde in 0.1 M phosphate buffer (PB; pH 7.4) overnight at 4 °C. The next day, it was washed three times with 0.1 M PB and cut along the sagittal plane using a vibratome (Leica VT2100S). Serial parasagittal sections (50 mm thick) were cryoprotected in a 30% sucrose solution in PB and stored in ethylene glycol/glycerol at 20 °C until they were analyzed. Regarding the other hemisphere, the hippocampus was rapidly dissected on ice and frozen in liquid nitrogen for other studies.

### Immunofluorescence techniques

For immunofluorescence experiments, free-floating serial sections (50-μm thick) were first rinsed in PB and then pre-incubated for 2 h in PB with 0.25% Triton-X100 and 3% normal serum of the species in which the secondary antibodies were raised (Normal Goat Serum / Normal Donkey Serum, Invitrogen, Thermo Scientific). To study proliferation and neurogenesis, serial sections were previously pre-treated in 2 M HCl for 15 min at 30 °C. Subsequently, brain sections were incubated for 24 h at 4 °C in the same pre-incubation stock solution (PB + Triton + Serum) containing different combinations of the following primary antibodies: ab6326 (Abcam) for CldU; AB2253 (Sigma Aldrich) for Doublecortin; ab8898 (Abcam) for Histone H3 (tri methyl K9); ab78517 (Abcam) for Histone H4 (dimethyl K20, tri methyl K20); TYR1336 Antibody (PhosphoSolution) for NMDA GluN2B Subunit (p1516-1336); ab32423 (Abcam) for brain lipid-binding protein (Blbp); 33D3 (Epigentek) for 5-methylcytosine (5mC); 39791 (Active-Motif) for 5-Hydromethylcytosine (5hmC) antibody; L1516 (Merck) for Lectin from *Wisteria floribunda* (WFA); ABN78 (Millipore) for NeuN and IF03L (Calbiochem) for GFAP.

After rinsing in the sections in PB, they were incubated for 2 h at room temperature in the appropriate combinations of Alexa 488-594-647-conjugated anti-mouse/rabbit/guinea pig or rat IgG secondary antibodies (1:500; Molecular Probes, Eugene, OR, USA). Sections were also labelled with the nuclear stain DAPI (4,6-diamidino-2-phenylindole; Sigma, St. Louis, MO, USA). In all cases, the sections used were those closest to the injection area and thus were in the same positions.

We obtained stitched image stacks from the whole DG in the hippocampus. These were recorded at 1-μm intervals through separate channels with a 20× lens for the analysis of neurogenesis, the extracellular matrix (ECM) and the expression of Sox2 in astrocytes (GFAP) and neurons (NeuN), and at 0.45/0.6-μm intervals through separate channels with a 40× lens for the analysis of the *N*-methyl-d-aspartate (NMDA) GluN2B subunit, and DNA and histone methylation, respectively (Nikon A1R confocal microscope, NA 0.75, refraction index 1, image resolution: 1024 × 1024 pixels). The same range of z-slices was obtained from each slide in each experiment. Adobe Photoshop (CS4) software was used to build the figures.

### Immunohistochemical quantifications

Dcx/Blbp/CldU-immunoreactive cells were quantified by counting the number of cells in each DG, distinguishing between the SGZ, granular cell layer (GCL), and the adjacent hilus region, in four consecutive medial brain sections. For perineuronal nets (PNN) of the ECM, positive units labeled with WFA were counted in the DG (including the GCL and hilus) from three consecutive medial brain sections.

For the study of Sox2 differential expression in neurons (NeuN-immunoreactive cells) or astrocytes (GFAP-immunoreactive cells), the co-expression of each Sox2 + cell in the dentate gyrus with NeuN or GFAP was counted in the DG (including the GCL and hilus) from three consecutive medial brain sections in every three-dimensional stack. To determine the density of different cells, the different layers of DG were traced on the DAPI channel of the z projection of each confocal stack of images, and the area of this structure was measured using the freehand drawing tool in Fiji. This area was multiplied by the stack thickness to calculate the reference volume. The number of positive cells was divided by the reference volume, and the density (number of cells/mm^3^) of cells was calculated.

For the 5mC, 5hmC, H3K9me3, and H4K20me3 antibodies, confocal settings (laser intensity, gain, pinhole) were kept constant for all images, which were captured in the same confocal session. Using the DAPI channel, the whole GCL was previously selected as an ROI. An invariant subtract background and threshold was then set in Fiji for each stack projection. The area above the threshold was measured in Fiji and the mean fluorescence in the ROI was calculated and compared between vehicle and folate experimental groups.

GluN2B, Sox2 and GFAP immunoreactivity was quantified with the ImageJ program by measuring the percentage of area occupied. The immunoreactivity of GluN2B protein was measured in the molecular layer of the DG, while the immunoreactivity of YF Sox2 and GFAP was assessed in the hilus region, SGZ, and GCL. An invariant subtract background and threshold were also set in Fiji for each stack projection.

### Nuclear fractionation of cultured human cells and western blotting

The method to fractionate the cell nucleus has been reported previously^49^, and the presence of FRα-binding peptide in the nuclear fraction was demonstrated by Western blot analysis using the antibody against FRα1-binding peptide (Thermo Fisher, ref: PA5-101588).

SK-N-SH cells were treated with folate (0.5 or 1 mM), FRα-binding peptide (0.5 or 1 mM), or Dulbecco's Modified Eagle Medium (DMEM) for 30 min at 37 °C. Total cell lysate or nuclear fraction was obtained as previously reported^19^. Whole cell lysate or cell nuclear pellets of each experiment were then quantified by the BCA protein assay^50^. Samples were separated on 10% SDS-PAGE and electrophoretically transferred to a nitrocellulose membrane (Schleicher & Schuell GmbH). The membrane was blocked by incubation with 5% semi-fat dried milk in PBS and 0.1% Tween 20 (PBS), followed by a 1-h incubation at room temperature with the primary antibody in PBSM. The following primary antibody dilutions were used: anti-folate receptor alpha 1 (1/300; Thermofisher, ref: PA5-101588) for nuclear fraction; and anti-Sox2 (1/1000; R&D systems, ref: AF2018) for total cell lysate. Histone3 (1:1000; UPSTATE#05-499) and Lamin B1 (1;1000; Santa Cruz SC-377000) respectively, were used as loading control. After three washes, the membrane was incubated with a horseradish peroxidase-anti-rabbit Ig conjugate (DAKO), followed by several washes in PBS-Tween 20. The membrane was then incubated for 1 min in Western Lightning reagents (PerkinElmer Life Sciences). Blots were quantified using the EPSON Perfection 1660 scanner and the Image J software.

### Behavioral tests

Open field (OF) and novel object recognition (NOR) tests were performed as described previously^8^. Locomotor activity, as well as anxiety and depression-like behavior, was evaluated using the OF test during 10 min session. In brief, on the first day, the mice were placed individually in a 45 × 45 cm plastic box with vertical opaque walls for 10 min. Each session was recorded, and data such as distance, average speed, time immobile, and time on the center square of the box were analyzed with ANY-maze software. The next day NOR test was performed. The mice were placed in the same box for 5 min and allowed to explore two identical objects: A and B (two black rook chess pieces). The two objects were placed on the long axis of the box, each 13 cm from the end of the box. After each exposure, the objects and the box were wiped with 70% ethanol to eliminate odors that could potentially condition the behavior of successive mice. Two hours after the familiarization trial, each mouse was released into the box with the same object previously used (object A) and a new one (object C, a tower of colored plastic pieces), instead of object B (short-term memory test). Object C was placed in the same position as object B in the familiarization trial. The mice were given 5 min to explore the box. Five days later, the mice were released into the box again, with object A in the same position and another new object (object D, a falcon tube filled with wood chips) in the same position as object C in the previous test. Again, the animals were allowed 5 min to explore the box (long-term memory test).

The memory index (MI) was used to measure recognition memory performance. The MI was defined as the ratio of time spent exploring or the number of entries in the new object (tC or tD) to the time spent exploring both objects (tA + tC or tA + tD) (MI = [tC/(tA + tC)] × 100 and [tD/(tA + tD)] × 100). ANY-maze software was used to calculate the time mice spent exploring the different objects.

To assess spatial memory, a Y maze test based on published protocols with modifications^51^ was used. This test is based on the innate curiosity of rodents to explore novel areas. First, the mice were placed into one of the arms of a black Y-maze apparatus, comprising three plastic arms forming a “Y” shape. They were then allowed to explore the maze for 10 min, with one of the arms closed (training trial). After 1 h, the mice were returned to the same arm of the Y maze (start arm) and were allowed to explore all three arms of the maze for 5 min. The number of entries into and the time spent in each arm were registered on video recordings and analyzed by ANY-maze software. We compared the percentage of time spent in the “novel” arm during the whole trial as a measure of spatial memory performance.

### Synthesis of the FRα-binding peptide

To mimic the cognitive results produced by intrahippocampal injection of folate but activating only the FRα-binding peptide, we used a synthetic FRα-binding peptide (purity of 90%) with the following sequence: cys–thr–val–arg–thr–ser–ala–glu–cys (CTVRTSAEC)^19^. This peptide was prepared by the company Abyntek (Vizcaya, Spain).

### Statistical analysis for immunohistochemical and behavior assessments

The data are presented as mean values ± SEM. Statistical analyses were performed using GraphPad Prism8. The experiments, as well as the acquisition and analysis of all data, were conducted in a randomized order by investigators who were blinded to the experimental conditions. To compare the two experimental groups (mice injected with vehicle and with folate or FRα-binding peptide), data were previously tested for normality using Shapiro–Wilk test and an unpaired Student’s t test was carried out. In SK-N-SH cells, a two-way ANOVA with Tukey's multiple comparisons test was conducted to study differences in Sox2 and FR-alpha protein expression among various treatments at different concentrations. A 95% confidence interval was applied for statistical comparisons.

### Supplementary Information

Supplementary Information.

## Data Availability

The data that support the findings of this study are openly available in figshare.com at https://figshare.com/articles/dataset/All_dataset/22277158.
