# Supplementary material for: Role of folate receptor α in the partial rejuvenation of dentate gyrus cells: Improvement of cognitive function in 21-month-old aged mice
Source: Sci Rep. 2024 Mar 22;14:6915. doi: 10.1038/s41598-024-57095-x (PMC10960019; doi:10.1038/s41598-024-57095-x)
Supplement: Supplementary file 1 — Supplementary Information. [file 41598_2024_57095_MOESM1_ESM.docx]

**Role of folate receptor α in the partial rejuvenation of dentate gyrus cells. Improvement of cognitive function in 21-month-old aged mice**

Antón-Fernández A^1^; Cuadros R^1,2^; Peinado-Cahuchola R^1^; Hernández F^1^; Avila J^1,2*^

^1^ Centro de Biología Molecular "Severo Ochoa", CSIC/UAM, Universidad Autónoma de Madrid, Cantoblanco, 28049 Madrid, Spain.

^2^ Center for Networked Biomedical Research on Neurodegenerative Diseases (CIBERNED), Madrid, Spain.

^*^ Correspondence to: Jesús Avila, Centro de Biología Molecular "Severo Ochoa", CSIC/UAM, Universidad Autónoma de Madrid, Cantoblanco, 28049 Madrid, Spain. Electronic address: javila@cbm.csic.es.

Running title: Folate receptor role in rejuvenation of DG cells.

**Abstract**

Neuronal aging may be, in part, related to a change in DNA methylation. Thus, methyl donors, like folate and methionine, may play a role in cognitive changes associated to neuronal aging. To test the role of these metabolites, we performed stereotaxic microinjection of these molecules into the dentate gyrus (DG) of aged mice (an average age of 21 month). Folate, but not S-Adenosyl-Methionine (SAM), enhances cognition in aged mice. In the presence of folate, we observed partial rejuvenation of DG cells, characterized by the expression of juvenile genes or reorganization of extracellular matrix.

Here, we have also tried to identify the mechanism independent of DNA methylation, that involve folate effects on cognition. Our analyses indicated that folate binds to folate receptor α (FRα) and, upon folate binding, FRα is transported to cell nucleus, where it is acting as transcription factor for expressing genes like SOX2 or GluN2B.

In this work, we report that a FRα binding peptide also replicates the folate effect on cognition, in aged mice. Our data suggest that such effect is not sex-dependent. Thus, we propose the use of this peptide to improve cognition since it lacks of folate-mediated side effects. The use of synthetic FRα binding peptides emerge as a future strategy for the study of brain rejuvenation.

**Results**

1. Effect of folate on adult neurogenesis in the dentate gyrus

As in our previous work ^1^, we tested the effect of folate on neuronal precursor cells, studying changes in brain lipid-binding protein (blbp) (**SI 3a**) and doublecortin-immunoreactive (ir) cell densities. Also, to label dividing cells, mice were injected intraperitoneally with CldU a week before perfusion, thus allowing the study of newborn 1-week-old cells in the SGZ. A single injection of folate into the hippocampus led to only a subtle increase in newborn CldU-ir cells (**SI 3b**) and in 1-week-old neurons (Dcx and CldU double-positive cells) (**SI 3c**) in the SGZ. Although the increase was not statistically significant, the tendency was similar to that found for YF ^1^. Furthermore, we found no differences in Blbp-ir (**SI 3a**) or doublecortin-ir cell densities (**SI 3d**) upon folate injection. Therefore, folate had no significant effect on adult hippocampal neurogenesis (AHN).

Indeed, in our previous work ^1^, a major YF-dependent change was found only in developmentally generated neurons compared to neurons raised in adult neurogenesis in the hippocampus. Thus, we subsequently tested the effect of folate on developmentally generated cells in the DG, looking at specific markers like changes in histone or DNA methylation.

2. Impact of folate on the methylation of histone and DNA in hippocampal neurons

The expression of aging-related genes correlates, as suggested by the heterochromatin loss aging model ^2^, with increasing alterations of chromatin located in heterochromatin regions. In this regard, we examined whether folate injection modifies the degree of histone methylation, namely at positions H3K9me3 and H4K20me3, which are present mainly in heterochromatin regions. The presence of folate 14 days post-injection did not modify the level of methylation at H3K9me3 (**SI 4a, b**) or H4K20me3 (**SI 4c, d**).

Our findings suggest that a single injection of folate might not be enough to promote reliable changes in histone methylation levels. Therefore, we tested whether folate affects DNA methylation. Significant increases in 5mC were observed in the granular neuronal layer (P=0.0132) of the DG where the injection was performed and it also found a lower increase at CA3 (P-value=0.05) and CA1 (P-value=0.06) (**SI 5d, e**). Regarding 5hmC, we also tested its levels. No significant changes were found in hippocampal neuronal layers (**SI 6**).

**Figures**





**Supplementary Figure 1. Experimental timeline and intracerebral injections. (a)** Schematic illustration of the timeline of experiments performed herein. **(b)** Illustration (modified from Paxinos, George, and Keith B.J. Franklin. The mouse brain in stereotaxic coordinates: hardcover edition. Access Online via Elsevier, 2001) of anatomical localization of the bilateral mono-dose injections (red plot) of different metabolites (SAM, and folate) and FRα-binding peptide or vehicle solution performed in the dentate gyrus (Anteroposterior -2 mm; Mediolateral ±1.4 mm; Dorsoventral -2.2 mm) of wild-type mice (male and female were randomized into experimental groups). Representative examples of trial hippocampal injections with dextran tracer (in red) performed before the start of the treatments are shown at the bottom. Note the trajectory of the 5ul Hamilton syringe, which only reaches the hilus of the DG.

**Supplementary Figure 2. Multiple folate effects, unique Frα**-**binding peptide effects.** (**a**) As indicated in the text, folate may play a role in several pathways. (**b**) FRα-binding peptide serves only to regulate the expression of specific genes. In this regard, FRα-binding peptide emerges as a more suitable therapeutic agent for the regulation of gene expression.





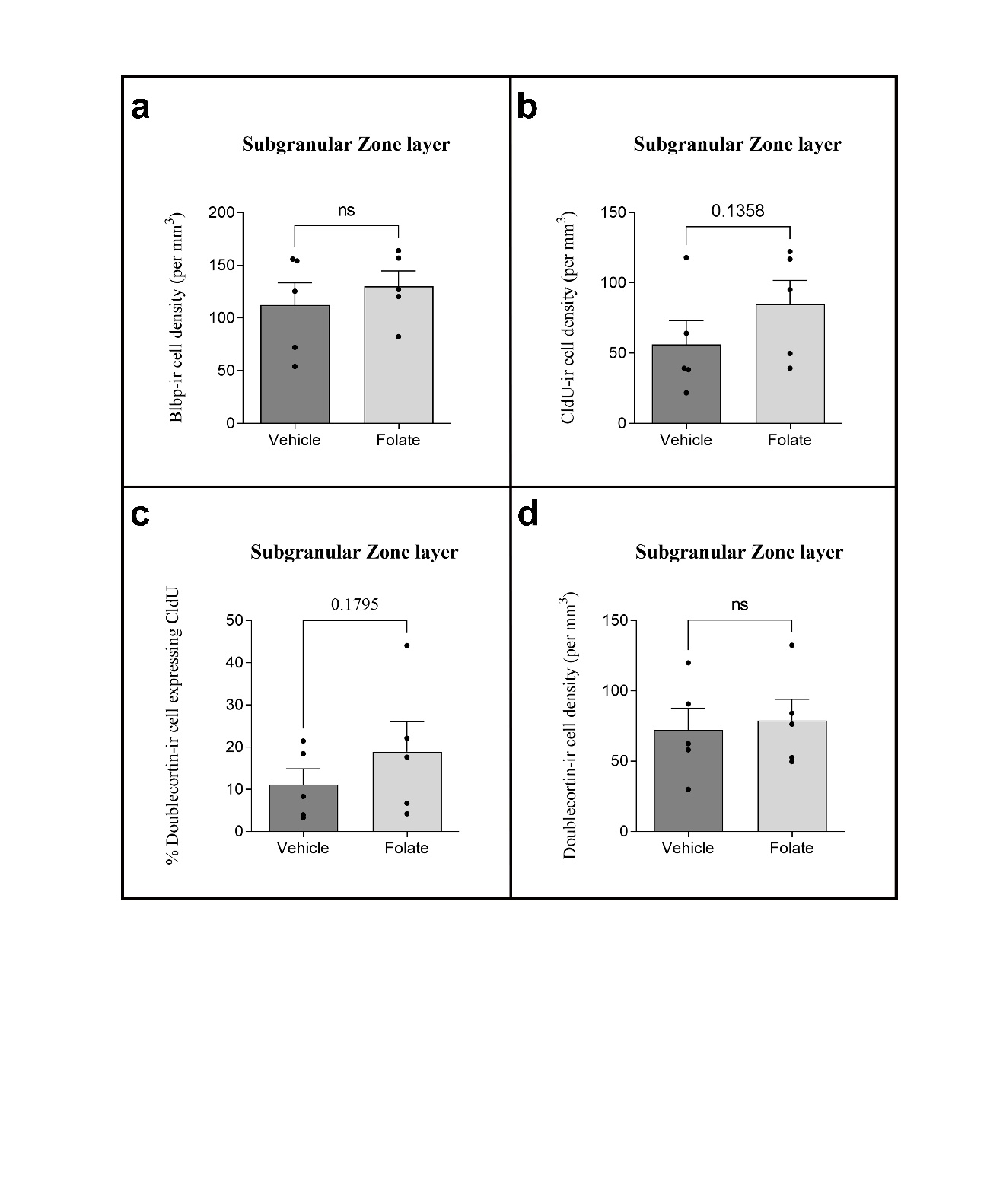


**Supplementary Figure 3. Effect of folate on the expression of adult neurogenesis markers**. (**a**) Histogram showing the density of Blbp-ir cells per mm^3^ in the subgranular zone (SGZ) of the dentate gyrus. Single-dose treatment with folate did not cause statistically significant changes in early neural precursors in the SGZ (t=0.0931, df=8). (**b**) Histogram showing the density of CldU-ir cells per mm^3^ in the SGZ. Single-dose treatment with folate did not lead to statistically significant changes in the generation of new cells in the SGZ(t=1.181, df=8). (**c**) Histogram showing the percentage of doublecortin-ir cells that were also CldU-immunoreactive in the SGZ. Single-dose treatment with folate did not cause statistically significant changes in the density of new 2-week-old neurons in the SGZ (t=0.9730, df=8). (**d**) Histogram showing the density of doublecortin-ir cells per mm^3^ in the SGZ. Single-dose treatment with folate did not bring about statistically significant changes in the density of young neurons in the SGZ(t=0.3176, df=8). (Mean ± SEM; ns: not significant differences; Student`s t-test).





**Supplementary Figure 4. Levels of H3K9me3 and H4K20me3. Effect of folate**. (**a**) Representative stitched confocal images of the H3K9me3 marker (in red) in the dentate gyrus (DG). Inset also shows the DAPI signal (in blue) to identify the DG structure. (**b**) Histogram showing the mean intensity of the histone methylation signal (t=0.2917, df=8 and t=0.1471, df=8 in DG and CA1, respectively). The effect of the absence (dark gray) or presence (light gray) of folate is shown. (**c**) Representative stitched confocal images of the H4K20me3 marker (in green) in the DG. Inset also shows the DAPI signal (in blue) to identify the DG structure. (**d**) Histogram showing the mean intensity of the histone methylation signal (t=0.4826, df=8 and t=0.5932, df=8, in DG and CA1, respectively). The effect of the absence (dark gray) or presence (light gray) of folate is shown. (Mean ± SEM; ns: not significant differences; Student`s t-test). Scale bar, 100 µm.





**Supplementary Figure 5. Effect of folate on DNA methylation of hippocampal neurons.** Representative stitched confocal images of 5-methycytosine (5mC) marker (in green) in the dentate gyrus of vehicle-treated (**a**) or folate-treated (**b**) mice. The corresponding high magnifications of the granular cell layer of a and b (yellow squares) are shown. Heterochromatin clusters of 5mC immunoreactivity can be observed in both magnifications below a and b. **(c**), (**d**) and (**e**) histograms showing the mean intensity of the 5mC signal in the granular cell layer of DG (t=2.717, df=8) and in pyramidal cell layer in CA3 (t=1.829, df=8) and CA1 (t=1.737, df=8) hippocampal regions. The effect of the absence (dark gray) or presence (light gray) of folate is shown (mean ± SEM, *p<0.05. Student’s test; ns: not significant differences).





**Supplementary Figure 6. Levels of 5hmC. Effect of folate**. (**a**) Representative stitched confocal images of the 5-hydroxymethylcytosine (5hmC) marker (in red) in the dentate gyrus (DG) of vehicle-treated and folate-treated mice. (**b**) Histogram showing mean intensity from 5mC signal of the granular cell layer of the DG (t=0.3579, df=8). The effect of the absence (dark gray) or presence (light gray) of folate is shown. (Mean ± SD; *p<0.05, Student`s t-test; ns: not significant differences). Scale bar, 100 µm.





**Supplementary Figure 7. Presence of the FRα** **and Sox2 on SK-N-SH cells by addition of folate or the FRα-binding peptide.** (**a**) Boshnjaku et al. ^3^described that, upon folate addition, FRα (which appears as two bands around 42 kD and 38 kD) can be internalized into the cytoplasm (~42 kD band) or the nucleus (~38 kD band). We studied the nuclear localization of FRα after using two concentrations of folate and FRα-binding peptide (0.5 and 1 mM) on SK-N-SH human neuroblastoma cells. Our results show the presence of a ~38 Kd band in the nuclear fraction in the presence or absence of folate or FRα-binding peptide. Shown in (**b**) are the relative protein expression level of FRα under folate and FRα-binding peptide in comparison to the control group, following a 30-minute incubation period. (**c**) The addition of folate or FRα-binding peptide increases the expression of Sox2 protein in SK-N-SH cells. Shown in (**d**) are the relative protein expression level of Sox2 under 0.5mM and 1mM folate and FRα-binding peptide in comparison to the control group, following a 30-minute incubation period. (Mean ± SEM; *p<0.05; ***p<0.0001. Two-way Anova with Tukey`s multiple comparisons test; (F(2,6)=5.769) and (F(2,6)=29.63), respectively in b and d).





**Supplementary Figure 8. Levels of 5mC and 5hmC. Effect of the FRα-binding peptide**. Histograms showing mean intensity of the 5mC (**a**) and 5hmC (**b**) signals of the granular cell layer of the dentate gyrus (DG). The effect of the absence (dark gray) or presence (light gray) of the FRα-binding peptide (2.5 mg/ml) is shown. No significant effect on the DNA methylation status of the DG was found (t=1.029, df=10 and t=1.217, df=10). (Mean ± SEM; *p<0.05, Student`s t-test; ns: no significant differences).

**References**

1 Rodriguez-Matellan, A., Alcazar, N., Hernandez, F., Serrano, M. & Avila, J. In Vivo Reprogramming Ameliorates Aging Features in Dentate Gyrus Cells and Improves Memory in Mice. *Stem Cell Reports* **15**, 1056-1066 (2020). <https://doi.org:10.1016/j.stemcr.2020.09.010>

2 Villeponteau, B. The heterochromatin loss model of aging. *Exp Gerontol* **32**, 383-394 (1997). <https://doi.org:10.1016/s0531-5565(96)00155-6>

3 Boshnjaku, V. *et al.* Nuclear localization of folate receptor alpha: a new role as a transcription factor. *Sci Rep* **2**, 980 (2012). <https://doi.org:10.1038/srep00980>
